# The Interplay Between Personal Identity and Social Identity Among Vocational High School Students: A Three-Wave Longitudinal Study

**DOI:** 10.1007/s10964-024-02073-9

**Published:** 2024-09-20

**Authors:** Kazumi Sugimura, Shogo Hihara, Kai Hatano, Tomotaka Umemura, Elisabetta Crocetti

**Affiliations:** 1 Graduate School of Humanities and Social Sciences, Hiroshima University, 1-1-1 Kagamiyama, Higashihiroshima, Hiroshima 739-8524 Japan; 2 Faculty of Business Administration, Matsuyama University, 4-2 Bunkyo-cho, Matsuyama, Ehime 790-8578 Japan; 3 Graduate School of Sustainable System Science, Osaka Metropolitan University, 1-1 Gakuen-cho, Naka-ku, Sakai, Osaka 599-8531 Japan; 4 Department of Psychology, Alma Mater Studiorum University of Bologna, Viale Berti Pichat 5, Cesena, FC 47521 Italy

**Keywords:** Personal identity, Social identity, Adolescence, Vocational high school, Japan, Longitudinal

## Abstract

Although identity research has predominantly focused on college-bound adolescents, it has largely neglected vocational high school students who enter the job market immediately after graduation. Furthermore, most studies have concentrated on personal identity and have overlooked the concurrent development of social identity. This study examined the relationship between adolescents’ personal and social identities over three years of vocational high school. The participants were 4,264 vocational high school students in Japan (Time 1: 46.44% girls; *M*_age_ = 15.78). Using a random intercept cross-lagged panel model, the results indicated that within-person increases in social identity predicted increases in personal identity one year later. These findings highlight the importance of social identity as a valuable resource for personal identity development among vocational high school students, a group underrepresented in identity research.

## Introduction

During adolescence, individuals form their *personal identity*, that is, define “who I am” as a unique existence and find an appropriate place or niche in their living environments (Erikson, 1968). This task of personal identity formation is inherently embedded in social contexts (Crocetti et al., 2023). Adolescents belong to and identify with various social groups, such as friends and schools. Through such identifications, adolescents develop their *social identity*, that is, the part of their self-concept stemming from their belonging to social groups, along with the value and emotional significance attached to those memberships (Tajfel, 1978). Thus, social identity is intricately interwoven with adolescents’ personal identity development. Although the dynamic interplay between personal and social identity has attracted increasing attention (Crocetti et al., 2018), researchers have overlooked identity development in adolescents attending vocational high schools (Sugimura et al., 2023). Focusing on those adolescents could elucidate the interplay between personal and social identity, which has so far been less known. Consequently, this study was conducted with adolescents in a non-Western country (Japan) and focused on a relatively understudied population, vocational high school students.

### Personal Identity

Personal identity refers to an individual’s subjective sense of self-sameness and continuity, a central task during adolescence (Erikson, 1968). Adolescence is marked by dramatic physical, cognitive, and social changes that compel adolescents to reconstruct their childhood self-perceptions and explore their goals, values, and beliefs as they transition into adulthood. A healthy personal identity is crucial for several developmental outcomes (Crocetti, 2017). Indeed, longitudinal research has demonstrated that positive developmental trajectories of identity were associated with life satisfaction during adolescence and young adulthood (Hatano et al., 2022). Furthermore, a firm sense of identity during the high school years predicted successful adjustment to work or tertiary education in young adulthood (Sugimura et al., 2023). Longitudinal research has also indicated that personal identity typically matures systematically across adolescence (see Branje et al., 2021, for a review), when individuals consolidate a sense of who they are (Bogaerts et al., 2021) and make significant decisions in multiple aspects of their lives, such as education and friendships (Klimstra et al., 2010).

In Erikson’s (1968) psychosocial developmental theory, adolescence is a life stage characterized by the tension between identity synthesis and identity confusion. Identity synthesis refers to the extent of a coherent sense of self over time and across situations, whereas identity confusion refers to a fragmented, changeable, and confused sense of self. From the Eriksonian perspective (Schwartz et al., 2009), healthy identity development is represented as finding an optimal balance between synthesis and confusion—that is, a predominance of synthesis over confusion. Thus, identity synthesis and identity confusion are not on a continuum; they are separate but coexist within the same person (Marcia, 2002). Previous studies have supported Erikson’s (1968) tenet. Factor analyses have shown that the two aspects are not on the opposite ends of a spectrum; they are rather distinct but related components of identity (Hatano et al., 2022). Furthermore, these two aspects have shown different developmental trajectories during adolescence and emerging adulthood (Hatano et al., 2022) and have been differently associated with diverse facets of youth adjustment (Sugimura et al., 2016).

### Social Identity

From a developmental social-psychological perspective (Crocetti et al., 2023), personal identity is inherently embedded in social contexts where adolescents identify with various social groups (e.g., family and school; Bronfenbrenner, 1979). Through these memberships, adolescents develop their own social identity. Social identity refers to “an individual’s self-concept which derives from his [or her] knowledge of his [or her] membership of a social group (or groups) together with the value and emotional significance attached to that membership” (Tajfel, 1978, p. 63). Positive social identity is crucial for enhancing an individual’s health and well-being (Jetten et al., 2017). For instance, longitudinal studies have found that positive changes in school identification predicted a decrease in bullying behavior among high school students (Turner et al., 2014). Additionally, multiple social identifications (e.g., as students and family members) positively predicted self-esteem among early and middle adolescents (Benish-Weisman et al., 2015). Longitudinal research has also outlined the fundamental developmental trajectory of social identification, suggesting that identification with close groups, with whom adolescents have concrete daily experiences, progressively drives identification with more abstract groups (Crocetti et al., 2023). Specifically, identifying with classmates affected the extent to which adolescents identify with humanity as the most inclusive superordinate group (Albarello et al., 2021). This developmental trajectory was confirmed in another study, demonstrating that social identification with proximal groups (i.e., family and classmates) facilitated the development of a more abstract level of social identification with ethnic and national groups (Karataş et al., 2023).

Social identity comprises two major levels of identification with groups: *identification with group* and *identification with group members* (Karasawa, 1991; see also Jackson & Smith, 1999; Prentice et al., 2006). Identification with group is defined as “knowledge of membership … and … value and emotional significance attached to the membership” (e.g., a sense of belonging and pride in one’s group; Karasawa, 1991, p. 295). In contrast, identification with group members is defined as “emotional attachment to in-group members and social influence from these peer members” (e.g., a sense of attachment to one’s group members; Karasawa, 1991, p. 295). Factor analyses with samples of specialized training college students have supported the distinction of these two components of social identity (Karasawa, 1991).

### Interplay Between Personal Identity and Social Identity in the School Context

Theoretically, school is a pivotal place where the interplay between personal and social identity is facilitated by curricula and circumstances (Crocetti et al., 2018). Personal identity is assumed to be a predictor of social identity. A clear sense of personal identity (i.e., identity synthesis) represents the sense of feeling that one knows where one is headed and could thus facilitate students’ future career plans. This would increase the students’ sense of membership in schools or professional courses. Moreover, adolescents with a synthesized sense of identity tend to have good interpersonal relationships (Kroger & Marcia, 2011); these friendly relationships help them find a place in the group and reinforce their sense of membership with classmates or friends. Conversely, social identity is assumed to predict personal identity. Developmentally, group identification is a precursor to a personal sense of identity during adolescence (Erikson, 1968; see also, Newman & Newman, 2001). Moreover, in school, students identify themselves with a variety of groups (e.g., clubs, classmates, and friends), and these groups provide them with numerous concrete social experiences. Everyday experiences such as social comparison and feedback from peers may help students define who one is as a unique existence and feed one’s personal sense of identity (Crocetti, 2018), and sometimes even the sacrifice of individual freedom in a group can make members realize the importance of a sense of individuality (Lois, 1999).

Thus far, empirical evidence testing these theoretical assumptions has been limited. Three studies have used adolescents from different contexts and examined the direction of associations between personal and social identity at the between-person and within-person levels, respectively. Between-person models provide insights into the rank-order stability within a group over time (Papp, 2004). For instance, when considering social and personal identity, a between-person model can determine whether adolescents who score higher on social identity than their peers also score higher on personal identity compared to their peers one year later. Therefore, it is crucial to evaluate adolescent scores in relation to the average scores of all adolescents in between-person models. In contrast, within-person models elucidate the dynamic relationship between two variables within an individual (Papp, 2004). For instance, a within-person model can reveal whether an increase in an adolescent’s social identity score leads to an increase in the same adolescent’s personal identity score. Thus, within-person models require the examination of adolescents’ scores in relation to their own average score. A study conducted at the between-person level mainly indicated that social identification (i.e., identification with peer groups) positively predicted interpersonal identity but not educational identity among Italian adolescents (Albarello et al., 2018). Additionally, at the between-person level, social identity represented by ethnic identity generally negatively predicted personal identity among immigrant adolescents in Greece; however, the finding might vary according to immigrant generation (i.e., the first and second generation groups; Mastrotheodoros et al., 2021). At the within-person level, a study with ethnic minority adolescents in Italy indicated no links between personal and social identity (Crocetti et al., 2024). These studies have suggested that the links between personal and social identity may depend on the level of analysis and the contexts (e.g., immigrant contexts) in which adolescents live.

To further understand the connection between personal and social identity, it is crucial to consider adolescents in diverse contexts (Sugimura, 2020). Previous studies have primarily focused on Western countries, leaving a gap in research on non-Western countries. Additionally, how personal and social identity intertwine in vocational high schools, where the emphasis lies on professional training rather than academic achievement, remains unexplored (Verhoeven et al., 2019). To gain a more comprehensive understanding of this dynamic, this study was conducted in a non-Western country (Japan) by focusing on an understudied population: vocational high school students.

### The Context of Vocational High Schools in Japan

Identity development in Japanese youth is characterized by instability and uncertainty, distinguishing it from the patterns observed in Western youth (Sugimura, 2020). Longitudinal studies have shown that from early to middle adolescence (i.e., junior high and high school years), identity synthesis remained higher than confusion. However, from late adolescence to young adulthood (i.e., university years and beyond), confusion tended to be higher than synthesis (Hatano et al., 2022). This trend contrasts with that observed among Western youth, who generally showed an increasing sense of identity synthesis and decreasing identity confusion during adolescence and young adulthood (Bogaerts et al., 2021, for Belgium youth; see Meeus, 2023, for a review of literature showing a pattern of identity maturation). The high sense of confusion among Japanese youth has been interpreted as a reflection of their difficulty in successfully transitioning to a changing society that increasingly values individualization and individuation. Japanese youth struggle to align the abilities, knowledge, and skills they acquire in school with societal expectations, particularly those of the industrial world (Arnett et al., 2014).

Focusing on vocational high schools in Japan is appropriate for investigating the interplay between personal and social identities in the context of a less smooth transition to society. Vocational high school students account for 17.1% of all high school students in Japan. In vocational high schools, 46.7% of graduates enter the labor market, 25.6% enroll in special training schools, and 25.2% enroll in universities; conversely, in academic-oriented high schools, these rates are 6.3%, 18.3%, and 70.3%, respectively (Ministry of Education, Culture, Sports, Science and Technology, 2023b). Although the rate of vocational high school students who enroll in universities is relatively low, Japanese vocational high schools have become increasingly academically oriented toward higher education since the 1990s owing to the popularization of higher education and the declining birthrate. This shift seems more pronounced in Japan than in the U.S. or Europe (Horiuchi et al., 2006). Moreover, Japan is a society in which educational backgrounds are highly valued; hence, 84% of students continue their education after high school to seek specialized knowledge and skills (Ministry of Education, Culture, Sports, Science and Technology, 2023b). Furthermore, university graduates in Japan tend to have higher lifetime earnings than high school graduates (The Japan Institute for Labour Policy and Training, 2023). Therefore, continuing education is considered socially desirable compared to only graduating from high school. In this context, students attending vocational high schools often encounter specific challenges in connecting their personal and social aspects of self in the process of identity formation (Sugimura et al., 2023). They face the task of personal and social identity formation earlier than their peers in academic-oriented high schools, where most students enroll in universities. Thus, they are urged to align their abilities, knowledge, and skills with societal expectations within three years of the school term. This task could be difficult for students who are less aware of their own interests or who do not conform well to the social groups to which they belong. Focusing on vocational high schools in Japan could add to the understanding of how adolescents develop their personal and social identity facets in the context of such specific challenges.

Japanese vocational high schools have two remarkable features. First, in the context of Japanese schools, courses (e.g., engineering, agriculture, business studies, and homemaking) serve as important group labels with which students identify. While similar labels like subjects and majors refer to course contents, in Japanese vocational high schools, courses refer to groups of people who take the same majors. Vocational high school students and those around them (e.g., teachers and parents) regard students in the same course as a group of people studying the same specialization, and label these students with the name of the course. The importance attached to the course a student takes determines the student’s reputation and has an advantageous effect on employment after graduation (Horiuchi et al., 2006). Each course in each school has its distinctive features and is valued for the distinctive activities it offers. Students are strongly aware of their membership in a course and the value and significance attached to it and therefore incorporate that recognition to enhance their pride and self-esteem by belonging to that group^1^. Moreover, the curriculum in Japanese vocational high schools is designed not only to foster professional knowledge and skills but also to provide comprehensive human education that fosters work ethic and cultivates esthetic and moral sentiments (Ministry of Education, Culture, Sports, Science and Technology, 2023a). Socialization in school, therefore, is viewed as a crucial educational goal. Indeed, vocational high schools offer various social activities and experiences at the course level, including athletic meetings, school festivals, and field trips. This cohesive curriculum not only fosters self-categorization among students in terms of their professional aspirations and aligns closely with their future careers, but it also strengthens students’ attachment to their courses. Therefore, this study focused on *identification with course* as a salient element of identification with group.

Second, in Japanese vocational high schools, relationships with classmates are likely to be close; hence, identification with classmates means emotional attachment to and being influenced by best friends. Previous studies on social identity have given distinctive meanings to social identifications with classmates and groups of friends. In various cultural contexts, classmates interact on a daily basis at school, but classmates are not necessarily actively chosen by themselves but passively assigned by schools; conversely, the group of friends consists of mutually chosen peers, which can be outside of school, with whom individuals spend a considerable portion of their leisure time (Albarello et al., 2018; see also, Crocetti et al., 2024). In Japan, however, classmates and the group of friends or best friends overlap. Similar to Italian adolescents, classmates are not chosen; however, Japanese adolescents choose close friends from their classmates and spend a substantial part of their time with them both in and outside of school. For instance, they spend breaks between classes and lunch time, participate in extracurricular activities (e.g., club activities), and enjoy leisure time outside of school together. Indeed, a national survey indicated that 90% of high school students find their best friends within the school (Children and Families Agency, 2020). Thus, for Japanese adolescents including vocational high school students, building a high-quality peer relationship means being successful at making best friends in the groups of classmates at school (Hatano et al., 2020). Therefore, this study focused on *identification with classmates* as a salient element of identification with group members.

## The Current Study

To examine the intertwined nature of multiple and multilayered aspects of identity (i.e., two components of personal identity and two levels of social identity), understanding how adolescents develop their identities in their complex social reality is important. Although personal identity and social identity are theoretically and empirically intertwined, how they are interrelated among vocational high schools from a non-Western context is still poorly known. This study aimed to address this gap by longitudinally examining the interplay between personal identity (i.e., identity synthesis and identity confusion) and social identity (i.e., identification with course and identification with classmates) over time, focusing on vocational high school students in Japan. Two hypotheses were put forth. First, at the between-person level, personal identity and social identity would be associated with each other throughout three years of high school. Specifically, identity synthesis would be positively, and identity confusion negatively, associated with identification with course and with classmates. Second, at the within-person level, personal identity and social identity would have bidirectional relations. Specifically, identity synthesis would positively, and identity confusion negatively, predict these two aspects of social identity. In addition, both identification with course and identification with classmates would positively predict identity synthesis and negatively predict identity confusion.

## Methods

### Participants

Participants were 4,264 Japanese adolescents involved in the first, second, and third waves of the longitudinal research project “Identity DEvelopment in Vocational high school students (IDEV)” (Sugimura et al., 2023). The IDEV examines identity development among adolescents from 28 vocational high schools located in a midwestern part of Japan. At the beginning of this study, all participants were first-year students at vocational high schools (studying, e.g., engineering, agriculture, nursing). The adolescents participated over the course of their three years of high school, as is typical in the Japanese educational system. This study was conducted three assessments at one-year intervals. Specifically, 4,261 adolescents (52.80% boys, 46.44% girls, 0.75% missing; *M*_age_ = 15.78 years, *SD*_age_ = 0.45 years) participated at Time 1 (T1). At Time 2 (T2), 4,155 adolescents participated (52.97% boys, 46.23% girls, 0.79% missing; *M*_age_ = 16.72 years, *SD*_age_ = 0.46 years). At Time 3 (T3), 4,017 adolescents participated (52.73% boys, 46.45% girls, 0.82% missing; *M*_age_ = 17.69 years, *SD*_age_ = 0.47 years).

Most adolescents provided data during all measurement waves (91.58%), and the remaining ones (8.42%) missed one or two measurement waves. The rates of missing data at the item level were 2.25%, 4.37%, 7.53%, at T1, T2, and T3, respectively. The results of Little’s (1988) Missing Completely at Random test indicated a normed χ^2^ (χ^2^/*df* = 54,891.148/50,389) of 1.09, which suggested that the present data were probably missing at random. Thus, all participants in the present sample (*N* = 4,264) were analyzed, and the Full Information Maximum Likelihood (FIML) procedure in M*plus* was used to deal with missing data (Kelloway, 2015).

### Procedure

This longitudinal research project was approved by the ethics committee of Hiroshima University, Japan. The school principals of the 28 vocational high schools gave permission for the questionnaires to be administered in schools. Additionally, adolescents and their parents received written information about the present study and provided active consent before data collection. Across all measurement waves, teachers distributed the questionnaires in the paper-and-pencil version, which the adolescents completed during class time. Participants were assigned their own personal codes at T1 to match their responses across the three time points. All adolescents voluntarily participated in this longitudinal study and could choose not to complete the questionnaires at any time. The data and materials are available on the OSF (https://osf.io/vx3ds/).

### Measures

Across all measurement waves, participants completed the same questionnaires, including sociodemographic information (e.g., age, biological sex, and study major) and measures of personal and social identity.

#### Personal identity

Personal identity was assessed using the Erikson Psychosocial Stage Inventory (EPSI; Rosenthal et al., 1981; Japanese validation by Hatano et al., 2014). This scale consists of 12 items assessing identity synthesis (e.g., “I have got a clear idea of what I want to be”) and identity confusion (e.g., “I change my opinion of myself a lot”), with six items for each subcomponent. All items were rated on a 5-point Likert-type scale, ranging from 1 (*completely untrue*) to 5 (*completely true*). One identity synthesis item, “I have a strong sense of what it means to be female/male,” was not used as it was considered undesirable from a gender perspective (Sugimura et al., 2023). Cronbach’s α coefficients across the three measurement waves were 0.68–0.74 for identity synthesis and 0.72–0.77 for identity confusion.

#### Social identity

Social identity was measured using the Group Identification Scale (GIS; Karasawa, 1991), which was originally developed and validated for use with Japanese-speaking people. This scale includes 13 items—eight for identification with group and five for identification with group members^2^. Consistent with the purpose of this study, items for each of these subcomponents were modified to assess identification with adolescents’ own course (e.g., “Is your sense of belonging to this department/course strong or weak?”) and identification with their classmates (e.g., “Are there many students in this department/course who influenced your thoughts and behaviors?”), respectively. All items were rated on a 5-point Likert-type scale ranging from 1 to 5 (see more details of this measurement in the Supplementary Materials). Cronbach’s α coefficients across the three measurement waves were 0.85–0.89 for identification with course and 0.74–0.81 for identification with classmates.

### Analytic Strategy

All the analyses were conducted using M*plus* 8.6 with the maximum likelihood robust estimator (Satorra & Bentler, 2001). As preliminary analyses, descriptive statistics and bivariate correlations of the study variables were calculated. Furthermore, confirmatory factor analyses (CFAs) were performed for both personal (identity synthesis and identity confusion) and social (identification with course and identification with classmates) identity at each measurement wave. Additionally, longitudinal measurement invariance was tested for personal and social identity across T1–T3. The specific procedure for examining CFAs and longitudinal measurement invariance is explained in the Supplementary Materials.

As main analyses, random intercept cross-lagged panel models (RI-CLPM; Hamaker et al., 2015) were tested to examine the longitudinal associations of personal identity and social identity. The models estimated random intercepts for the study variables, which indicate stable between-person differences among the participants. Within-person differences are the intraindividual fluctuation relative to each participant’s trait-level scores. The RI-CLPM can examine within-person associations among the study variables by separating them from between-person associations. The models estimated (a) cross-lagged paths (e.g., prospective path from identity synthesis at T1 to identification with course at T2), (b) stability paths (e.g., autoregressive path between identity synthesis at T1 and T2), (c) correlations at T1, and (d) correlated changes at T2–T3 among all study variables. In line with the recommendation of Orth et al. (2021), time-invariance tests were examined for all cross-lagged and stability paths. Specifically, the model fit indices of an unconstrained baseline model (M1) was compared with those of a model fixing all the cross-lagged paths to be invariant across time (T1 → T2 and T2 → T3; M2), a model fixing all the stability paths to be invariant across time (T1 → T2 and T2 → T3; M3), and a model fixing all the cross-lagged and stability paths to be invariant across time (T1 → T2 and T2 → T3; M4). Additionally, as ancillary sensitivity analyses, the longitudinal associations between personal identity and social identity were examined, while including adolescents’ sex and major as covariates.

The models were evaluated using the Comparative Fit Index (CFI), Root Mean Square Error of Approximation (RMSEA), and Standardized Root Mean Square Residual (SRMR). In the CFI, values higher than 0.900 indicate acceptable fit indices, while values higher than 0.950 suggest excellent fit indices. As for the RMSEA and SRMR, values less than 0.080 represent acceptable fit indices, while values less than 0.050 indicate excellent fit indices (Byrne, 2012). Additionally, to compare the fit indices of the different models, the changes in Satorra-Bentler scaled chi-square (ΔS-Bχ^2^; Satorra & Bentler, 2001), the CFI (ΔCFI), and the RMSEA (ΔRMSEA) were evaluated. Models were determined to be significantly different when at least two of the three criteria were met: statistically significant ΔS-Bχ^2^ at *p* < 0.050, ΔCFI ≤ −0.010, and ΔRMSEA ≥ 0.015 (Chen, 2007).

## Results

### Preliminary Analyses

Table 1 presents descriptive statistics and bivariate correlations of the study variables. As reported in Fig. S1 and Table S3 of the Supplementary Materials, two-factor models for personal identity (i.e., identity synthesis and identity confusion) and social identity (i.e., identification with course and identification with classmates) fitted well with the data across T1–T3. Furthermore, as shown in Table S4, the longitudinal measurement invariance tests indicated that full metric invariance for both measures of personal and social identity could be established. Thus, it was possible to conduct the main analyses (i.e., RI-CLPM).

**Table 1:** Means, standard deviations, and correlations among the study variables

Variables	*M*	*SD*	2.	3.	4.	5.	6.	7.	8.	9.	10.	11.	12.	13.
1. Sex	0.47	0.50	−0.05**	−0.01	−0.04*	−0.05**	−0.04**	−0.06***	0.10***	0.07***	0.07***	0.08***	0.10***	0.07***
Personal identity														
2. Identity synthesis T1	3.12	0.68	–	0.56***	0.48***	−0.32***	−0.30***	−0.26***	0.23***	0.19***	0.16***	0.35***	0.24***	0.22***
3. Identity synthesis T2	3.16	0.68		–	0.56***	−0.32***	−0.35***	−0.31***	0.21***	0.26***	0.20***	0.26***	0.31***	0.25***
4. Identity synthesis T3	3.35	0.70			–	−0.30***	−0.34***	−0.34***	0.19***	0.22***	0.26***	0.21***	0.24***	0.30***
5. Identity confusion T1	2.69	0.68				–	0.59***	0.51***	−0.19***	−0.17***	−0.16***	−0.18***	−0.15***	−0.15***
6. Identity confusion T2	2.74	0.71					–	0.59***	−0.17***	−0.21***	−0.18***	−0.15***	−0.19***	−0.17***
7. identity confusion T3	2.61	0.72						–	−0.15***	−0.15***	−0.16***	−0.14***	−0.14***	−0.15***
Social identity														
8. Identification with course T1	3.28	0.73							–	0.50***	0.46***	0.51***	0.38***	0.37***
9. Identification with course T2	3.32	0.76								–	0.60***	0.36***	0.60***	0.45***
10. Identification with course T3	3.49	0.77									–	0.32***	0.44***	0.63***
11. Identification with classmates T1	3.25	0.68										–	0.61***	0.53***
12. Identification with classmates T2	3.23	0.74											–	0.66***
13. Identification with classmates T3	3.32	0.79												–

Sex: 0 = boy, 1 = girl; *T* time, *M* mean, *SD* standard deviation.

*p < 0.05; **p < 0.01; ***p < 0.001.

### Associations Between Personal Identity and Social Identity

Model comparisons (see Table 2) indicated that time invariance could be established for both cross-lagged paths (M2) and stability paths (M3). Furthermore, the most parsimonious model (M4) with all the cross-lagged paths and stability paths constrained to be time-invariant was retained as the final one (see Table 2). Between-person level correlations, standardized within-person level path coefficients, and within-time correlations are shown in Tables 3–5, respectively.

**Table 2:** Model fit indices and comparisons for random intercept cross-lagged panel models

Models	Model fit indices					Model comparisons					
	S-Bχ^2^	*df*	CFI	RMSEA [90% CI]	SRMR	Pairs	ΔS-Bχ^2^	Δ*df*	*p*	ΔCFI	ΔRMSEA
M1	23.07	6	0.999	0.026 [0.015, 0.037]	0.008						
M2	59.10	10	0.997	0.034 [0.026, 0.043]	0.021	M2–M1	36.78	4	<0.001	−0.002	0.012
M3	39.83	18	0.998	0.017 [0.010, 0.024]	0.013	M3–M1	16.96	12	0.151	−0.001	−0.009
**M4**	**65.71**	**22**	**0.997**	**0.022 [0.016, 0.028]**	**0.024**	**M4–M1**	**42.71**	**16**	**<0.001**	−**0.002**	−**0.004**

The accepted model is shown in bold.

*M1* unconstrained baseline model, *M2* model with all cross-lagged paths fixed to be time-invariant, *M3* model with all stability paths fixed to be time-invariant, *M4* model with all cross-lagged paths and stability paths fixed to be time-invariant, *S-Bχ*^2^ Satorra-Bentler chi-square value, *df* degree of freedom, *CFI* comparative fit index, *RMSEA* root mean square error of approximation, *SRMR* standardized root mean squared residual, *90%CI* 90% confidence interval, *Δ* change in parameter.

**Table 3:** Between-person associations of the random intercept cross-lagged panel analysis

Variables		2.	3.	4.
Personal identity				
1. Identity synthesis	*r*	−0.56***	0.34***	0.43***
	95% CI	[−0.62, −0.49]	[0.27, 0.41]	[0.37, 0.49]
2. Identity confusion	*r*	–	−0.33***	−0.31***
	95% CI	–	[−0.40, −0.25]	[−0.38, −0.23]
Social identity			–	
3. Identification with course	*r*		–	0.65***
	95% CI			[0.60, 0.70]
4. Identification with classmates	*r*			–
	95% CI			–

*95%* *CI* 95% confidence interval.

***p < 0.001.

**Table 4:** Standardized coefficients of the random intercept cross-lagged panel analysis

Explanatory variables		Outcome variables							
		Identity synthesis		Identity confusion		Identification with course		Identification with classmates	
		T1 → T2	T2 → T3	T1 → T2	T2 → T3	T1 → T2	T2 → T3	T1 → T2	T2 → T3
Personal identity									
Identity synthesis	β	0.16***	0.16***	−0.07*	−0.06*	0.03	0.04	0.01	0.01
	95% CI	[0.10, 0.22]	[0.09, 0.22]	[−0.12, −0.02]	[−0.11, −0.02]	[−0.01, 0.08]	[−0.01, 0.08]	[−0.03, 0.05]	[−0.03, 0.05]
Identity confusion	β	−0.10***	−0.10***	0.20***	0.22***	−0.03	−0.03	−0.00	−0.00
	95% CI	[−0.15, −0.05]	[−0.15, −0.05]	[0.15, 0.26]	[0.15, 0.28]	[−0.07, 0.02]	[−0.08, 0.02]	[−0.05, 0.04]	[−0.05, 0.04]
Social identity									
Identification with course	β	0.07**	0.08**	−0.03	−0.03	0.20***	0.24***	0.10***	0.11***
	95% CI	[0.02, 0.12]	[0.02, 0.13]	[−0.07, 0.02]	[−0.08, 0.02]	[0.15, 0.26]	[0.17, 0.31]	[0.06, 0.15]	[0.06, 0.16]
Identification with classmates	β	0.02	0.02	0.03	0.04	0.12***	0.15***	0.28***	0.34***
	95% CI	[−0.03, 0.07]	[−0.04, 0.08]	[−0.02, 0.08]	[−0.02, 0.10]	[0.07, 0.17]	[0.09, 0.21]	[0.23, 0.34]	[0.27, 0.40]

*95%* *CI* 95% confidence interval, *T* time.

*p < 0.05; **p < 0.01; ***p < 0.001.

**Table 5:** Within-person concurrent correlations and correlated changes of the random intercept cross-lagged panel analysis

Variables		Identity confusion			Identification with course			Identification with classmates		
		T1	T2	T3	T1	T2	T3	T1	T2	T3
Personal identity										
Identity synthesis	*r*	−0.11**	−0.15***	−0.11***	0.14***	0.19***	0.17***	0.27***	0.20***	0.17***
	95% CI	[−0.18, −0.04]	[−0.22, −0.09]	[−0.17, −0.05]	[0.08, 0.20]	[0.13, 0.25]	[0.12, 0.22]	[0.21, 0.33]	[0.14, 0.25]	[0.13, 0.22]
Identity confusion	*r*	–	–	–	−0.06	−0.12***	−0.04	−0.05	−0.12***	−0.02
	95% CI	–	–	–	[−0.12, 0.00]	[−0.18, −0.07]	[−0.08, 0.01]	[−0.13, 0.02]	[−0.17, −0.06]	[−0.07, 0.04]
Social identity										
Identification with course	*r*				–	–	–	0.39***	0.56***	0.55***
	95% CI				–	–	–	[0.34, 0.45]	[0.52, 0.60]	[0.52, 0.59]
Identification with classmates	*r*							–	–	–
	95% CI							–	–	–

*95%* *CI* 95% confidence interval, *T* time.

**p < 0.01; ***p < 0.001.

The results at the between-person level highlight correlations between personal and social identity. Identity synthesis was positively related to both identification with course and with classmates. Identity confusion was negatively correlated with both identification with course and with classmates. These findings support the first hypothesis, by which personal and social identity are intertwined at the between-person level throughout three years of vocational high school.

**Fig. 1:**
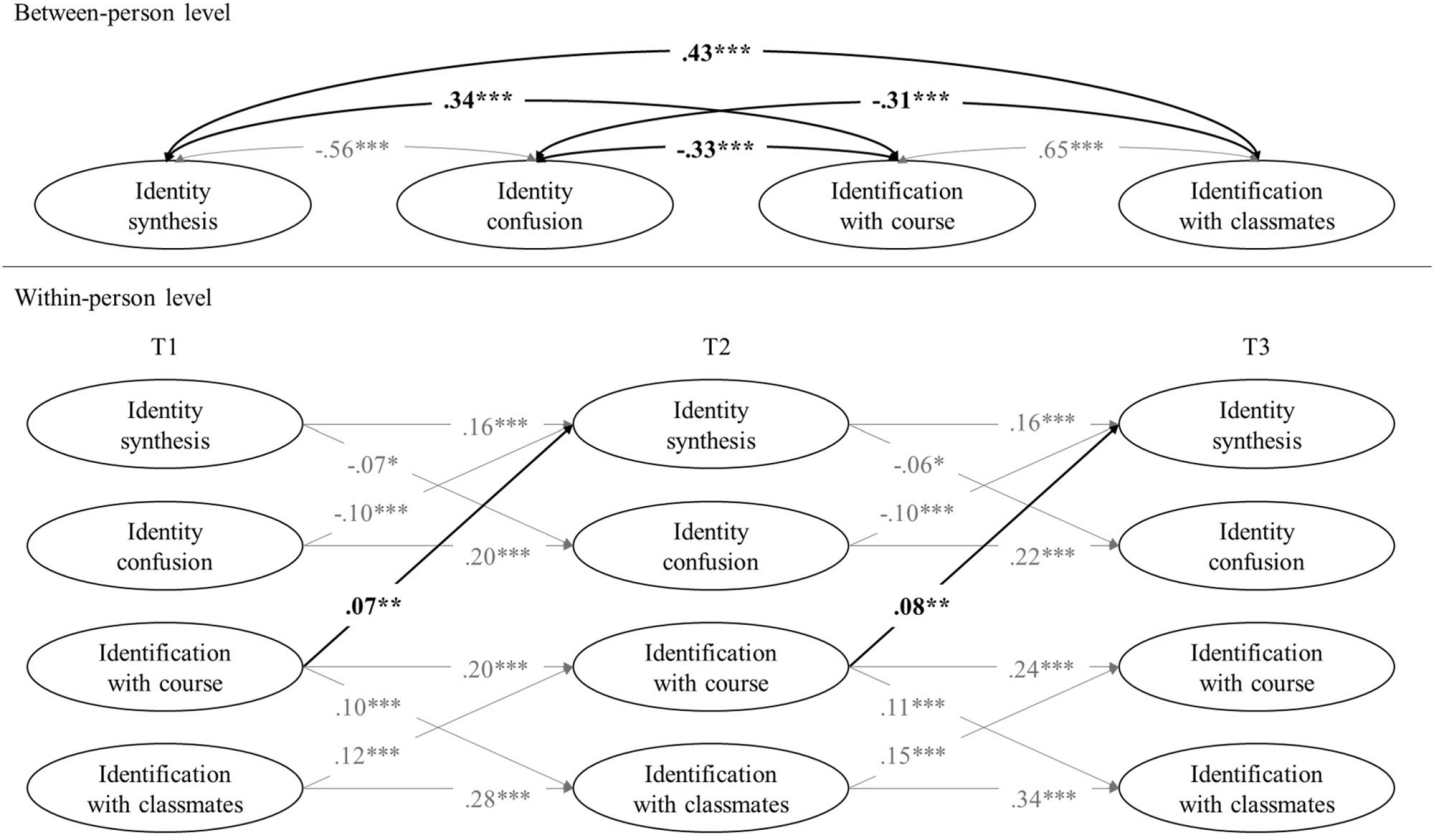
Correlations between between-person latent variables (upper figure) and standardized within-person cross-lagged and stability paths linking social identity and personal identity (lower figure). Bold paths indicate the statistically significant cross-construct associations, while gray paths indicate the statistically significant within-construct associations. *T* time. *p < 0.05; **p < 0.01; ***p < 0.001.

The results at the within-person level show one unidirectional cross-lagged effect from identification with course to identity synthesis. As reported in Fig. 1, when participants reported scores on identification with course higher than their own average, they increased the scores on identity synthesis the next year. Cross-lagged effects from identity synthesis and identity confusion to identification with course and with classmates were statistically insignificant. Additionally, cross-lagged effects from identification with classmates to identity synthesis and identity confusion were statistically insignificant. These findings suggest a predominance of unidirectional processes instead of the expected bidirectional effects.

Significant within-time correlations were also found at the within-person level. Identity synthesis was positively correlated with identification with course and with classmates across all measurement waves. These results indicate that when participants showed scores on identity synthesis higher than their average, they also reported higher scores on identification with course and identification with classmates at the same wave. Additionally, identity confusion was negatively related to identification with course and with classmates at T2.

### Sensitivity Analyses

For sensitivity analyses, sex and majors were controlled while estimating the longitudinal associations between personal and social identity. The results of the main analyses were replicated when the models included sex or majors as control variables (Tables S5–S7 and S9–S11). Nevertheless, girls reported lower identity synthesis at T1 and T3 and lower identity confusion across T1–T3 than boys (Table S8). Furthermore, they reported higher identification with the course and classmates across T1–T3 than boys. Regarding majors (Table S12), adolescents with people-oriented majors (homemaking, nursing, or social welfare; Kuhn & Wolters, 2022; Su et al., 2009) showed higher identity synthesis and lower identity confusion at T1 than those with things-oriented majors (information science, business studies, agriculture, engineering, or integrated studies). Additionally, adolescents with people-oriented majors exhibited higher identification with courses at T1 and identification with classmates across T1–T3 than others with things-oriented majors.

## Discussion

During adolescence, one engages in a crucial developmental task, that is, forming personal and social identity (Crocetti et al., 2018). The interplay between personal and social identity has attracted increased attention (e.g., Albarello et al., 2018). However, whether and how these two facets of identity are related among adolescents attending vocational high schools, who are urged to link their own skills and knowledge with social expectations, has not received due attention. To address this research gap, this study aimed to examine the relationship between personal and social identity by focusing on Japanese adolescents attending vocational high schools. The results of the random intercept cross-lagged panel model highlighted that, at the between-level, a close relationship existed among all components of personal and social identity. Further, at the within-person level, when adolescents reported above their own average on identification with course (i.e., social identity), they also reported later increased identity synthesis (i.e., personal identity). In addition, at the within-person level, within-time correlations revealed that identity synthesis was consistently associated with identification with course and classmates throughout the three-year term. Overall, these results provide novel insights into how personal and social identity develop among adolescents attending vocational high schools in Japan, who have been unexplored in previous studies.

### The Social Identity Roots of Personal Identity

At the between-level, identity synthesis was positively, and identity confusion negatively, correlated with identification with course and classmates, respectively, over a period of three years in vocational high schools. Thus, the first hypothesis was supported. This general trend of the results is consistent with findings from previous studies employing traditional cross-lagged panel models, although the groups adopted in such studies (e.g., identification with the group of friends; Albarello et al., 2018, for Italian adolescents) differed from the ones in the present study. These findings suggest that personal and social identity may be fundamentally, closely connected in both adolescents in Western (i.e., Italy) and non-Western (i.e., Japan) countries from diverse school contexts (i.e., academic-oriented, technical, or vocational high schools).

At the within-person level, further investigation into the effects of personal and social facets of identity demonstrated that the one cross-lagged effect was significant. Specifically, adolescents with higher scores on identification with course reported an increase (as compared to their own average level) in the scores of identity synthesis one year later. Conversely, personal identity did not predict social identity. Thus, although bidirectional effects were expected, the findings provided more support for unidirectional processes.

Among the above within-person cross-lagged results, it was noteworthy that increases in a vocational high school adolescent’s identification with a *course* led to increases in identity synthesis one year later. This is a novel finding as both the present study and previous research (Crocetti et al., 2024) focused on identification with *classmates* as social identity have found no association with personal identity. This novel knowledge about the effect of identification with a level of group (i.e., course) but not a level of individual relationships (i.e., classmates) in social identity suggests that identification with course is a particularly meaningful resource for personal identity in vocational high school students. The importance of identification with course may be especially salient among students in vocational high schools, where learning contents in respective courses are closely related to students’ future career; this clarifies what one is (i.e., who one is in society or the professional world), what kind of professional group one belongs to, and where one’s life is going. A strong sense of affiliation with a specific course brings students assurance about a firm sense of continuity between present (i.e., learning contents in vocational high schools) and future (i.e., professional career life). Additionally, the results may reflect the increasing academic orientation of Japanese vocational high schools (Horiuchi et al., 2006). For students interested in continuing their education after high school, a sense of belonging to a course may serve as an important resource for clarifying their future orientation, specifically their interest in enrolling in higher specialized education (i.e., universities).

The results also show that identity confusion was not associated with social identity, which suggests that confusion may be less susceptible to change in social identity among vocational high school students in Japan. One plausible reason is that, in Japanese vocational high schools, curriculums are strictly determined, and schools have a strong connection with local industries (Ministry of Education, Culture, Sports, Science and Technology, 2023a). Thus, students can foresee their future career relatively easily. Even if they become uninterested in their own courses, they may not immediately lose their way, which may prevent them from being confused about where they are going.

The finding that personal identity did not predict social identity implies that changes in the levels of a clear or fragmented sense of self may not necessarily affect changes in the levels of identification with course or classmates. This tendency supports the theoretical notion that social identity serves as the foundation of personal identity in adolescence (Newman & Newman, 2001). Furthermore, adolescents can define their overall sense of personal synthesis or confusion from the concrete experiences they have in their social environments. The results highlight that social identity may be a precursor of personal identity but not vice versa, at least among adolescents in vocational high schools.

As for the results of within-time correlations at the within-person level, interrelations between identity synthesis and identification with course and classmates were found throughout three years, but those between identity confusion and identification with course and classmates were reported only at T2. This suggests that identity synthesis, rather than confusion, is particularly important and intertwines substantially with adolescents’ social identity over three years of vocational high school.

Sensitivity analyses revealed that the model results were consistent between boys and girls, suggesting that social identity could be the root of personal identity among vocational high school adolescents of both sexes. Sex differences were observed: Boys scored higher on both personal identity dimensions than girls, while girls scored higher on both social identity dimensions than boys. The former finding, indicating that boys may be more sure about their personal identity than girls, is consistent with research showing that girls tend to explore in depth their identity commitments more than boys (e.g., De Lise et al., 2024). This active exploration can be a double-edged sword, leading them to question and doubt their current choices (Crocetti, 2017). The latter finding, indicating that girls report higher identification with social groups than boys confirmed the importance that girls attribute to close relationships (Morgan & Korobov, 2012). Furthermore, the finding that social identity predicted personal identity was consistent across majors. Differences between majors (Kuhn & Wolters, 2022; Su et al., 2009) were found in that students with people-oriented majors scored higher on identity synthesis, lower on identity confusion, and higher on both social identity dimensions than peers with things-oriented majors. This may reflect that people-oriented majors often include several practicums to develop interpersonal communication and skills to help others directly, leading to greater group cohesion and self-reflection.

In summary, this study provided a novel insight into the relationship between personal identity and social identity, highlighting the importance of the effect of identification with course on identity synthesis. Thus, among two major levels of social identity (e.g., Prentice et al., 2006), identification with a group itself rather than identification with the group members may be a key component in the development of personal and social facets of identity among adolescents in vocational high schools. This study has implications for multiple areas of studies. Regarding identity development research, this study is one of a few studies examining both personal identity and social identity. The novelty of the findings is the direction from social identity to personal identity which uniquely contributes to identity research. As an implication for research on vocational education, this study highlighted that for vocational high school students, identification with the course is more important than identification with classmates. This suggests that researchers and practitioners should prioritize students’ affiliation with and interest in their courses as crucial indicators of their healthy identity development. When guiding and counseling students, their attitudes toward learning contents such as practicums in the professional areas should not be overlooked throughout school years.

### Limitations and Future Research

This study has limitations that should be considered. First, the measurements of personal and social facets of identity relied solely on self-report measures. Self-report is a reliable method for assessing the facets of subjective sense of self in adolescence; however, incorporating other-report measures would enhance the robustness of the evidence. Second, this study focused on adolescents in vocational high schools in Japan. This sample was an appropriate choice to address the study aims; however, Japanese vocational high schools may be unique in their academic orientations. Therefore, the results of this study should be generalized with caution. Future research should include vocational high schools in other countries to test the replicability of these findings. Third, the internal consistency of the scales assessing personal identity (i.e., EPSI) was relatively low. Although the values were comparable to those reported in previous studies across different contexts such as Belgium (Bogaerts et al., 2021), the United States (Meca et al., 2017), and Japan (Sugimura et al., 2016), the low reliability of the scales calls for the development of a revised version of the EPSI with improved psychometric properties. Finally, the items assessing identification with the course were designed to measure students’ awareness of their membership and the extent to which they identified with the course as a group. However, some participants may have responded by primarily referring to their level of identification with course contents (e.g., subjects) rather than the course as a group of people. Future research should revise these items to focus more precisely on identifying courses as a group.

## Conclusion

Research has begun to address the interplay between personal and social identity; nevertheless, whether and how these two facets of identity are related among adolescents from non-Western countries attending non-academic-oriented high schools has so far remained unclear. To address this research gap, this study targeted vocational high school adolescents in Japan and examined the relationship between personal identity (identity synthesis and identity confusion) and social identity (identification with course and identification with classmates) during their three years of high school. The random intercept cross-lagged model revealed that an adolescent’s increased identification with course lead to increased identity synthesis one year later; thus, social identity predicted personal identity. Conversely, personal identity did not predict social identity. Further, within-time correlations at the within-person level demonstrated the importance of identity synthesis, rather than identity confusion, in the interplay between personal and social identity throughout the three years. Overall, this study contributes to the understanding of social identity as a valuable resource for personal identity development among adolescents in vocational high schools.

## Supplementary Information

Supplementary Materials

## Footnotes

1 For example, in one business studies course, the students worked with a local company to develop a new product using local inputs, which was highly appreciated by the locals. Belonging to and identifying with that group enhances students’ self-esteem and pride.

2 There are three versions of this scale with 7, 12, and 13 items (M. Karasawa, personal communication, July 27, 2018; Oishi, 2001). The present study used the 13-item version, which has been used in other studies as well (e.g., Nakashima et al., 2013).
