# Supplementary material for: The Interplay Between Personal Identity and Social Identity Among Vocational High School Students: A Three-Wave Longitudinal Study
Source: J Youth Adolesc. 2024 Sep 20;54(2):454–67. doi: 10.1007/s10964-024-02073-9 (PMC11807075; doi:10.1007/s10964-024-02073-9)
Supplement: Supplementary file 1 — Supplementary Materials [file 10964_2024_2073_MOESM1_ESM.docx]

**Supplementary Materials**

**The Interplay Between Personal Identity and Social Identity Among Vocational High School Students: A Three-Wave Longitudinal Study**

Kazumi Sugimura^1^, Shogo Hihara^2^, Kai Hatano^3^, Tomotaka Umemura^1^, and Elisabetta Crocetti^4^

^1^ Graduate School of Humanities and Social Sciences, Hiroshima University; 1-1-1 Kagamiyama, Higashihiroshima-city, Hiroshima 739-8524, Japan

^2^ Faculty of Business Administration, Matsuyama University, 4-2 Bunkyo-cho, Matsuyama-city, Ehime 790-8578, Japan

^3^ Graduate School of Sustainable System Science, Osaka Metropolitan University; 1-1 Gakuen-cho, Naka-ku, Sakai-city, Osaka 599-8531, Japan

^4^ Department of Psychology, Alma Mater Studiorum University of Bologna; Viale Berti Pichat 5, Cesena, FC 47521, Italy

**Author Note**

Correspondence concerning this article should be addressed to Kazumi Sugimura, Graduate School of Humanities and Social Sciences, Hiroshima University, 1-1-1 Kagamiyama, Higashihiroshima-city, Hiroshima 739-8524, Japan

Email: ksugimura@hiroshima-u.ac.jp

Phone: +81-82-424-6882

**Index**

**Sample Attrition Analyses…………………………………………………………………………..3**

**Items of Group Identification Scale……………………………………………………………….. 5**

**Confirmatory Factor Analyses and Tests of Longitudinal Measurement Invariance…………..9**

**Sensitivity Analyses………………………………………………………………………………..14**

**References………………………………………………………………………………………….23**

**Sample Attrition Analyses**

A total of 4,264 vocational high school students in Japan agreed to participate in the longitudinal research project “Identity DEvelopment in Vocational high school students (IDEV)”. Specifically, 4,261, 4,155, and 4,017 adolescents provided data across three years of their high school (T1, T2, and T3, respectively). To gain a better understanding of the sample attrition, adolescents who participated across T1–T3 (91.58%) were compared to those who did not participate in at least one measurement wave (8.42%). Regarding demographic variables, the results indicated no sex differences between adolescents who did or did not participate across all measurement waves (see Table S1; χ^2^(1, *N* = 4,231) = 0.15, *p* = .694, Cramer’s *V* = .01). Adolescents who participate across all measurement waves were younger than those who did not (see Table S2; *t* (4228) = 4.98, *p* < .001, Cohen’s *d* = .28 [.17, .38]). Regarding the main study variables (i.e., identity synthesis, identity confusion, identification with course, and identification with classmates measured across T1–T3), the results (Table S2) indicated that adolescents who participated across all measurement waves scored lower on identity synthesis at T2 (*t* (4139) = 2.72, *p* = .006, Cohen’s *d* = .18. [.05, .30]) and higher on identification with classmates at T3 (*t* (3958) = 3.05, *p* = .002, Cohen’s *d* = -.29 [-.48, -.10]), compared to adolescents who did not participate in at least one measurement wave. However, the effect size of these differences (Cohen’s *d*s) was small. This suggests that adolescents who did or did not provide data across T1–T3 were largely comparable, especially in terms of sex, age, and the main study variables.

Table S1

Cross tabulation of participation across T1–T3 and sex

|  | Participation across all measurement waves (%) | | Total (%) |
| --- | --- | --- | --- |
|  | Participation | Non-participation |  |
| Sex |  |  |  |
| Boys | 52.23 | 53.25 | 53.23 |
| Girls | 47.77 | 46.62 | 46.77 |

Table S2

Relations between participation across T1–T3 and the study variables

| Variables | Time | Participation | | *t*-values | *p* | Cohen’s *d* |
| --- | --- | --- | --- | --- | --- | --- |
|  |  | Participation across all time points  *M* (*SD*) | Non-Participation in at least one time point  *M* (*SD*) |  |  | [95% CI] |
| Age | T1 | 15.77 (0.44) | 15.89 (0.50) | *t* (4228) = 4.98 | < .001 | .28 [.17, .38] |
| Personal identity |  |  |  |  |  |  |
| Identity synthesis | T1 | 3.11 (0.68) | 3.17 (0.71) | *t* (4250) = 1.59 | .113 | .08 [-.02, .20] |
|  | T2 | 3.16 (0.68) | 3.28 (0.77) | *t* (4139) = 2.72 | .006 | .18 [.05, .30] |
|  | T3 | 3.35 (0.70) | 3.27 (0.70) | *t* (3999) = 1.14 | .256 | -.11 [-.30, .08] |
| Identity confusion | T1 | 2.69 (0.67) | 2.69 (0.70) | *t* (4255) = 0.26 | .798 | .01 [-.09, .12] |
|  | T2 | 2.74 (0.70) | 2.73 (0.77) | *t* (4142) = 0.12 | .904 | -.01 [-.14, .12] |
|  | T3 | 2.61 (0.72) | 2.67 (0.70) | *t* (4000) = 0.78 | .435 | .08 [-.11, .26] |
| Social identity |  |  |  |  |  |  |
| Identification with | T1 | 3.28 (0.72) | 3.27 (0.74) | *t* (4208) = 0.42 | .673 | -.02 [-.13, .09] |
| course | T2 | 3.32 (0.75) | 3.33 (0.89) | *t* (4111) = 0.22 | .830 | .01 [-.11, .14] |
|  | T3 | 3.46 (0.77) | 3.37 (0.71) | *t* (3953) = 1.22 | .224 | -.12 [-.31, .07] |
| Identification with | T1 | 3.26 (0.68) | 3.22 (0.71) | *t* (4224) = 1.07 | .287 | -.06 [-.17, .05] |
| classmates | T2 | 3.23 (0.73) | 3.21 (0.87) | *t* (4121) = 0.41 | .683 | -.03 [-.15, .10] |
|  | T3 | 3.33 (0.79) | 3.10 (0.82) | *t* (3958) = 3.05 | .002 | -.29 [-.48, -.10] |

*Note*. T = time; *M* = mean; *SD* = standard deviation; 95%CI = 95% conﬁdence interval.

**Items of Group Identification Scale**

**Japanese version**

以下には、現在あなたが所属している学科・コースをひとつの集団として捉えた項目が並べられています。それぞれの項目について、あなた自身の考えに最もよく当てはまる数字を１つ選んで、○で囲んでください。

1. 「あなたはこの学科・コースの生徒らしいタイプの人だね」と言われたとしたら、その表現は当たっている、つまり適切にあなたのことを表現していると思いますか。

全く適切でない　 　1　-　2　-　3　-　4　-　5　 　非常に適切である

1. あなたは他の人から、どの程度「この学科・コースの生徒らしいタイプの人」と思われていると思いますか。

全くこの学科・コースの生徒らしくない

　 　1　-　2　-　3　-　4　-　5

非常にこの学科・コースの生徒らしい

1. 「あなたはこの学科・コースの生徒らしいタイプの人だね」と言われたら、良い感じがしますか、それとも悪い感じがしますか。

非常に悪い感じ　 　1　-　2　-　3　-　4　-　5　 　非常に良い感じ

1. この学科・コースに対する所属意識は強い方ですか、弱い方ですか。

非常に弱い　 　1　-　2　-　3　-　4　-　5　 　非常に強い

1. あなたはこの学科・コースにプライドを感じますか。

全く感じない　 　1　-　2　-　3　-　4　-　5　 　非常に感じる

1. あなたにとって本当に大切な友人はこの学科・コース外・内のどちらに多くいますか。

この学科・コース外に多い　 　1　-　2　-　3　-　4　-　5　 　この学科・コース内に多い

1. あなたの考えや行動に影響を与えた人が、この学科・コース内にはどれくらいいますか。

全くいない　 　1　-　2　-　3　-　4　-　5　 　非常に多くいる

1. 「自分はこの学科・コースの人間なんだなあ」と実感することがありますか。

全くない　 　1　-　2　-　3　-　4　-　5　 　非常によくある

1. あなたは自己紹介するときや会話の中などで、自分がこの学科・コースに所属していることによくふれる方ですか、ふれない方ですか。

全くふれない　 　1　-　2　-　3　-　4　-　5　 　非常によくふれる

1. あなたはこの学科・コースにどのくらい愛着を感じていますか。

全く感じない　 　1　-　2　-　3　-　4　-　5　 　非常に強く感じる

1. あなたは、この学科・コースの人たちのことが好きな方ですか、嫌いな方ですか。

非常に嫌い　 　1　-　2　-　3　-　4　-　5　 　非常に好き

1. あなたは、この学科・コースのメンバーにどのくらい親近感を感じますか。

全く感じない　 　1　-　2　-　3　-　4　-　5　 　非常に強く感じる

1. あなたは性格や行動などが、この学科・コースの他のメンバーと似ていると思いますか、それとも違っていると思いますか。

非常に違っている　 　1　-　2　-　3　-　4　-　5　 　非常に似ている

注）項目1–5，8，9，10は学科・コースへの同一視を，項目6，7，11–13は学科・コースのメンバーへの同一視を測定している。

**English version**

Regarding the department/course you currently belong to as a group, the following questions ask about you. For each question, please choose the most appropriate number and circle it.

1. Would you think it is accurate if you were described as typical student of this department/course?

Not accurate at all　 　1　-　2　-　3　-　4　-　5　 　 Very accurate

1. To what extent do you think others regard you as “a typical student of this department/course”?

Not typical at all　 　1　-　2　-　3　-　4　-　5　 　 Very typical

1. Would you feel good if you were described as a typical student of this department/course?

Feel very bad　 　1　-　2　-　3　-　4　-　5　 　 Feel very good

1. Is your sense of belonging to this department/course strong or weak?

Very weak　 　1　-　2　-　3　-　4　-　5　 　 Very strong

1. Do you feel proud of this department/course?

Do not feel proud at all　 　1　-　2　-　3　-　4　-　5　　 Feel very proud

1. Where do most of your best friends come from, this department/course or not?

Many do not come from this department/course

1　-　2　-　3　-　4　-　5

Many come from this department/course

1. Are there many students in this department/course who influenced your thoughts and behaviors?

Not at all　 　1　-　2　-　3　-　4　-　5　 　 A great many

1. How often do you acknowledge the fact that you are a student of this department/course?

Not at all　 　1　-　2　-　3　-　4　-　5　 　 Very often

1. How often do you refer to the name of this department/course when you introduce yourself?

Not at all　 　1　-　2　-　3　-　4　-　5　 　 Very often

1. To what extent do you feel attached to this department/course?

Feel no attachment　 　1　-　2　-　3　-　4　-　5　 　 Feel very strong attachment

1. Do you like or dislike others in this department/course?

Dislike very much　 　1　-　2　-　3　-　4　-　5　 　 Like very much

1. To what extent do you feel close to other members of this department/course?

Do not feel close at all　 　1　-　2　-　3　-　4　-　5　 　 Feel very close

1. Do you think your personality or behavior is similar to that of others in this department/course?

Quite different　 　1　-　2　-　3　-　4　-　5　 　 Very similar

*Note*. Items 1–5, 8, 9, and 10 assess identification with department/course and items 6, 7, and 11–13 assess identification with the members of the department/course.

**Confirmatory Factor Analyses and Tests of Longitudinal Measurement Invariance**

**Confirmatory Factor Analysis**

A series of Confirmatory Factor Analyses (CFAs) were conducted to estimate the factor structure of the personal identity and social identity separately. Both models for personal identity and social identity were tested at each measurement wave. The model for personal identity included two-factors, where five and six items were loaded onto latent variables of identity synthesis and identity confusion, respectively. As reported in Table S3, the fit indices of personal identity models were acceptable across T1–T3. To improve the models, residual correlations between items were estimated based on modification indices. When residual correlations were included (see Figure S1), the models for personal identity fitted well across T1–T3.

The model for social identity was a two-factors model, where eight and five items were loaded onto latent variables of identification with course and identification with classmates, respectively. As shown in Table S3, the social identity models did not show good model fit indices across T1–T3. To improve the model fit indices, residual correlations were estimated on the basis of modification indices. When residual correlations were included (see Figure S1), the social identity models indicated good model fit indices across T1–T3.

Figure S1

Measurement models for personal identity (left figure) and social identity (right figure).


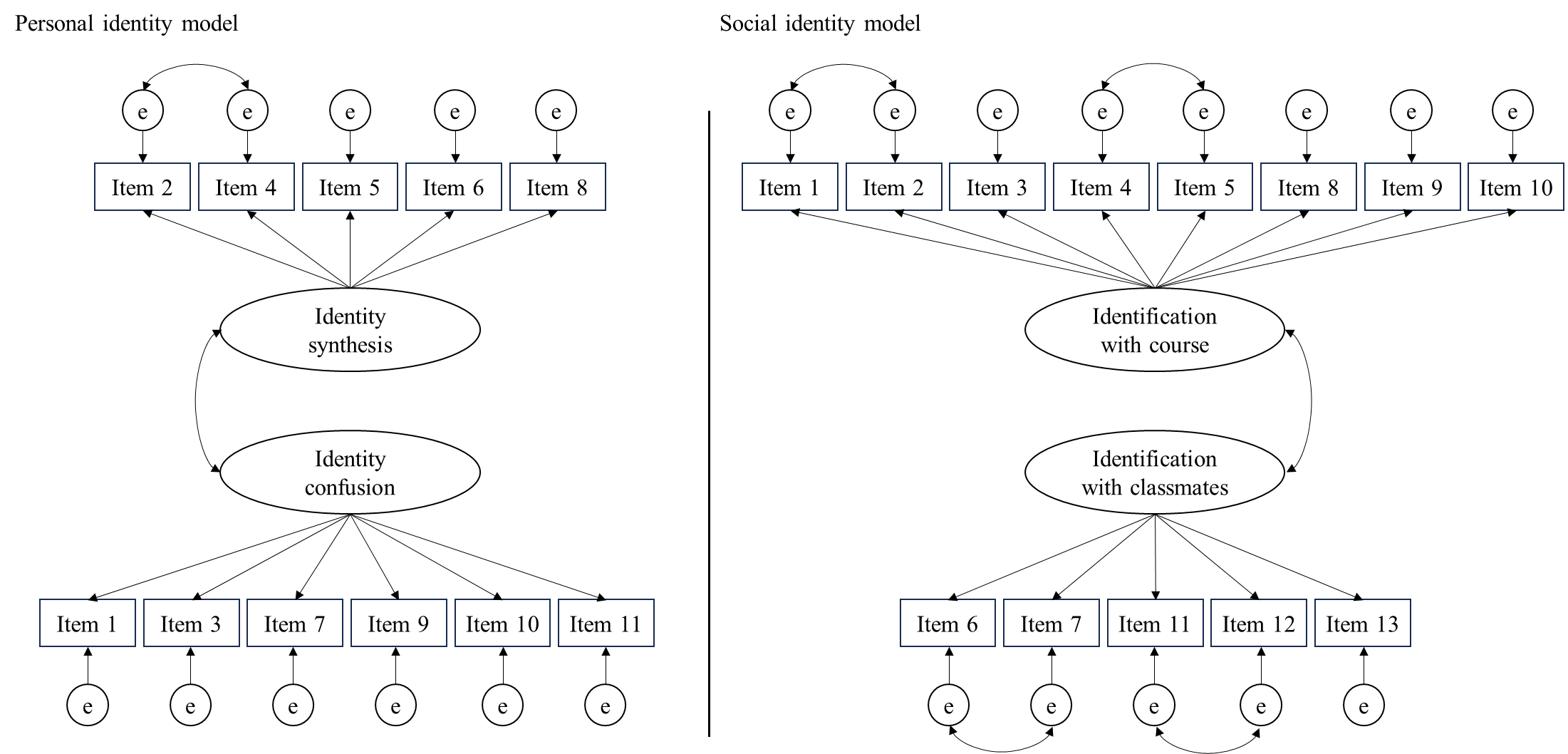


Table S3

Confirmatory factor analyses for personal identity and social identity

| Models | Time | Model fit indices | | | | |
| --- | --- | --- | --- | --- | --- | --- |
|  |  | χ^2^ | *df* | CFI | RMSEA [90%CI] | SRMR |
| Personal identity |  |  |  |  |  |  |
| Identity synthesis and identity confusion model | T1 | 740.45 | 43 | .892 | .062 [.058, .066] | .046 |
|  | T2 | 791.23 | 43 | .896 | .065 [.061, .069] | .046 |
|  | T3 | 653.95 | 43 | .920 | .060 [.056, .064] | .042 |
| Identity synthesis and identity confusion model with | T1 | 650.17 | 42 | .906 | .058 [.054, .062] | .044 |
| residual correlations | T2 | 713.82 | 42 | .907 | .062 [.058, .066] | .045 |
|  | T3 | 608.93 | 42 | .926 | .058 [.054, .062] | .041 |
| Social identity |  |  |  |  |  |  |
| Identification with course and identification with classmates | T1 | 1808.13 | 64 | .877 | .080 [.077, .083] | .052 |
| model | T2 | 2172.96 | 64 | .876 | .089 [.086, .093] | .054 |
|  | T3 | 2409.06 | 64 | .872 | .096 [.093, .099] | .055 |
| Identification with course and identification with classmates | T1 | 714.81 | 60 | .954 | .051 [.048, .054] | .033 |
| model with residual correlations | T2 | 866.43 | 60 | .953 | .057 [.054, .060] | .035 |
|  | T3 | 881.63 | 60 | .955 | .059 [.055, .062] | .033 |

*Note*. T = time; χ^2^ = chi-squared test; *df* = degree of freedom; CFI = comparative fit index; RMSEA = root mean square error of approximation and 90% conﬁdence interval; SRMR = standardized root mean squared residual.

**Longitudinal Measurement Invariance**

Longitudinal measurement invariance across the three measurement waves was tested for both personal identity and social identity. Residual correlations between the same items at different time points were estimated (e.g., the first item of synthesis at T1, T2, and T3). To test the longitudinal measurement invariance, the baseline (configural) model was compared to the metric model that entails the invariance of all factor loadings, indicating that adolescents attribute the same meaning to the latent variable of interest across the three measurement waves. When comparisons between models reported at least two out of three criteria, the longitudinal measurement invariance could not be established: ΔS-B*χ*^2^ significant at *p* < .05 (Satorra & Bentler, 2001), ΔCFI ≤ −.010, and ΔRMSEA ≥ .015 (Chen, 2007). As shown in Table S4, the results indicated that full metric invariance could be confirmed for both personal identity and social identity.

Table S4

Longitudinal measurement invariance of personal identity and social identity

| Models | Model fit indices | | | | |  | Model comparisons | | | | | |
| --- | --- | --- | --- | --- | --- | --- | --- | --- | --- | --- | --- | --- |
|  | χ^2^ | *df* | CFI | RMSEA [90%CI] | SRMR |  | Pairs | ΔS-Bχ^2^ | Δ*df* | *p* | ΔCFI | ΔRMSEA |
| Personal identity |  |  |  |  |  |  |  |  |  |  |  |  |
| M1. Configural | 2406.60 | 444 | .945 | .032 [.031, .033] | .042 |  |  |  |  |  |  |  |
| **M2. Full metric** | **2431.92** | **462** | **.944** | **.032 [.030, .033]** | **.042** |  | **M2-M1** | **21.74** | **18** | **.244** | **-.001** | **.000** |
| Social identity |  |  |  |  |  |  |  |  |  |  |  |  |
| M1. Configural | 4002.86 | 636 | .948 | .035 [.034, .036] | .035 |  |  |  |  |  |  |  |
| **M2. Full metric** | **4057.71** | **658** | **.948** | **.035 [.034, .036]** | **.036** |  | **M2-M1** | **44.42** | **22** | **.003** | **.000** | **.000** |

*Note*. M = model; χ^2^ = chi-squared test; *df* = degree of freedom; CFI = comparative fit index; RMSEA = root mean square error of approximation and 90% conﬁdence interval; SRMR = standardized root mean squared residual; *Δ* = Change in the parameter; The accepted model is shown in bold.

**Sensitivity Analyses**

Sensitivity analyses were conducted to examine the effects of sex and majors on the results of the main analyses. Specifically, longitudinal associations between personal identity (identity synthesis and identity confusion) and social identity (identification with course and identification with classmates) were estimated using a model that included adolescents’ sex and majors as control variables. Based on the existing literature on career development (Kuhn & Wolters, 2022; Su et al., 2009), adolescents’ majors were divided into things-oriented (information science, business studies, agriculture, engineering, or integrated studies) and people-oriented (homemaking, nursing, or social welfare). When sex was included as a control variable, the models were replicated (χ^2^ (22) = 66.64, *p* < .001, CFI = .997, RMSEA = .022 [.016, .028], SRMR = .023) (see Table S5–S7). As shown in Table S8, sex (0 = boy, 1 = girl) was negatively related to identity synthesis at T1 and T3, and identity confusion across T1–T3. Additionally, sex was positively related to identification with the course and classmates across T1 to T3. When the major was considered as a control variable, the models were replicated (χ^2^ (22) = 65.98, *p* < .001, CFI = .997, RMSEA = .022 [.016, .028], SRMR = .024) (see Tables S9–S11). As shown in Table S12, majors (0 = things-oriented, 1 = people-oriented) were positively correlated with identity synthesis and negatively correlated with identity confusion at T1. Additionally, majors were positively related to identification with the course at T1 and identification with classmates across T1–T3. Thus, the main results were confirmed when adolescent sex and major were controlled for.

Table S5

Sensitivity analysis for relations between personal identity and social identity at the between-person level, controlling for sex

| Variables |  | 2. | 3. | 4. |
| --- | --- | --- | --- | --- |
| Personal identity |  |  |  |  |
| 1. Identity synthesis | *r* | -.56^***^ | .35^***^ | .44^***^ |
|  | 95%CI | [-.63, -.50] | [.28, .42] | [.38, .50] |
| 2. Identity confusion | *r* | – | -.32^***^ | -.30^***^ |
|  | 95%CI | – | [-.40, -.25] | [-.37, -.23] |
| Social identity |  |  | – |  |
| 3. Identification with course | *r* |  | – | .65^***^ |
|  | 95%CI |  |  | [.60, .70] |
| 4. Identification with classmates | *r* |  |  | – |
|  | 95%CI |  |  | – |

*Note*. 95%CI = 95% conﬁdence interval.

^***^*p* < .001.

Table S6

Sensitivity analysis for longitudinal relations between personal identity and social identity at the within-person level, controlling for sex

| Explanatory variables |  | Outcome variables | | | | | | | |
| --- | --- | --- | --- | --- | --- | --- | --- | --- | --- |
|  |  | Identity synthesis | | Identity confusion | | Identification with course | | Identification with classmates | |
|  |  | T1→T2 | T2→T3 | T1→T2 | T2→T3 | T1→T2 | T2→T3 | T1→T2 | T2→T3 |
| Personal identity |  |  |  |  |  |  |  |  |  |
| Identity synthesis | β | .17^***^ | .16^***^ | -.07^**^ | -.07^**^ | .03 | .03 | .01 | .01 |
|  | 95%CI | [.11, .23] | [.10, .22] | [-.12, -.02] | [-.11, -.02] | [-.01, .08] | [-.01, .08] | [-.03, .06] | [-.03, .05] |
| Identity confusion | β | -.09^***^ | -.10^***^ | .21^***^ | .22^***^ | -.03 | -.03 | -.00 | -.00 |
|  | 95%CI | [-.14, -.05] | [-.15, -.05] | [.15, .27] | [.15, .29] | [-.07, .02] | [-.08, .02] | [-.04, .04] | [-.05, .04] |
| Social identity |  |  |  |  |  |  |  |  |  |
| Identification with course | β | .07^**^ | .07^**^ | -.02 | -.03 | .20^***^ | .24^***^ | .10^***^ | .10^***^ |
|  | 95%CI | [.02, .12] | [.02, .13] | [-.07, .02] | [-.08, .02] | [.15, .26] | [.17, .31] | [.05, .14] | [.06, .15] |
| Identification with classmates | β | .02 | .02 | .03 | .04 | .12^***^ | .15^***^ | .28^***^ | .34^***^ |
|  | 95%CI | [-.03, .07] | [-.04, .08] | [-.02, .08] | [-.02, .10] | [.07, .17] | [.09, .21] | [.23, .34] | [.27, .40] |

*Note*. 95%CI = 95% conﬁdence interval; T = time.

^**^*p* < .01; ^***^*p* < .001.

Table S7

Sensitivity analysis for within-time correlations and correlated changes between personal identity and social identity, controlling for sex

| Variables |  | Identity confusion | | | Identification with course | | | Identification with classmates | | |
| --- | --- | --- | --- | --- | --- | --- | --- | --- | --- | --- |
|  |  | T1 | T2 | T3 | T1 | T2 | T3 | T1 | T2 | T3 |
| Personal identity |  |  |  |  |  |  |  |  |  |  |
| Identity synthesis | *r* | -.11^**^ | -.15^***^ | -.11^***^ | .14^***^ | .19^***^ | .17^***^ | .28^***^ | .19^***^ | .17^***^ |
|  | 95%CI | [-.18, -.04] | [-.22, -.09] | [-.17, -.06] | [.08, .20] | [.13, .25] | [.12, .22] | [.22, .34] | [.14, .25] | [.12, .22] |
| Identity confusion | *r* | – | – | – | -.06 | -.12^***^ | -.04 | -.06 | -.11^***^ | -.02 |
|  | 95%CI | – | – | – | [-.12, .01] | [-.18, -.06] | [-.08, .01] | [-.13, .02] | [-.17, -.06] | [-.07, .03] |
| Social identity |  |  |  |  |  |  |  |  |  |  |
| Identification with course | *r* |  |  |  | – | – | – | .39^***^ | .56^***^ | .55^***^ |
|  | 95%CI |  |  |  | – | – | – | [.33, .45] | [.51, .60] | [.52, .59] |
| Identification with classmates | *r* |  |  |  |  |  |  | – | – | – |
|  | 95%CI |  |  |  |  |  |  | – | – | – |

*Note*. 95%CI = 95% conﬁdence interval; T = time.

^**^*p* < .01; ^***^*p* < .001.

Table S8

Associations of sex with personal identity and social identity

| Study variables |  | Sex (0 = boy, 1 = girl) | | |
| --- | --- | --- | --- | --- |
|  |  | T1 | T2 | T3 |
| Personal identity |  |  |  |  |
| Identity synthesis | β | -.07^**^ | -.02 | -.07^**^ |
|  | 95%CI | [-.11, -.03] | [-.06, .02] | [-.10, -.03] |
| Identity confusion | β | -.07^**^ | -.05^*^ | -.07^**^ |
|  | 95%CI | [-.12, -.03] | [-.08, -.01] | [-.10, -.03] |
| Social identity |  |  |  |  |
| Identification with course | β | .13^***^ | .05^**^ | .04^*^ |
|  | 95%CI | [.09, .17] | [.02, .09] | [.01, .08] |
| Identification with classmates | β | .12^***^ | .09^***^ | .04^*^ |
|  | 95%CI | [.07, .16] | [.05, .12] | [.01, .08] |

*Note*. 95%CI = 95% conﬁdence interval; T = time.

^*^*p* < .05; ^**^*p* < .01; ^***^*p* < .001.

Table S9

Sensitivity analysis for relations between personal identity and social identity at the between-person level, controlling for majors

| Variables |  | 2. | 3. | 4. |
| --- | --- | --- | --- | --- |
| Personal identity |  |  |  |  |
| 1. Identity synthesis | *r* | -.56^***^ | .34^***^ | .43^***^ |
|  | 95%CI | [-.62, -.49] | [.27, .41] | [.37, .50] |
| 2. Identity confusion | *r* | – | -.33^***^ | -.30^***^ |
|  | 95%CI | – | [-.40, -.25] | [-.38, -.23] |
| Social identity |  |  | – |  |
| 3. Identification with course | *r* |  | – | .64^***^ |
|  | 95%CI |  |  | [.59, .70] |
| 4. Identification with classmates | *r* |  |  | – |
|  | 95%CI |  |  | – |

*Note*. 95%CI = 95% conﬁdence interval.

^***^*p* < .001.

Table S10

Sensitivity analysis for longitudinal relations between personal identity and social identity at the within-person level, controlling for majors

| Explanatory variables |  | Outcome variables | | | | | | | |
| --- | --- | --- | --- | --- | --- | --- | --- | --- | --- |
|  |  | Identity synthesis | | Identity confusion | | Identification with course | | Identification with classmates | |
|  |  | T1→T2 | T2→T3 | T1→T2 | T2→T3 | T1→T2 | T2→T3 | T1→T2 | T2→T3 |
| Personal identity |  |  |  |  |  |  |  |  |  |
| Identity synthesis | β | .16^***^ | .15^***^ | -.06^*^ | -.06^*^ | .03 | .03 | .01 | .01 |
|  | 95%CI | [.10, .22] | [.09, .21] | [-.11, -.01] | [-.11, -.01] | [-.01, .08] | [-.01, .08] | [-.03, .05] | [-.03, .05] |
| Identity confusion | β | -.10^***^ | -.10^***^ | .20^***^ | .21^***^ | -.03 | -.03 | -.00 | -.00 |
|  | 95%CI | [-.15, -.05] | [-.15, -.05] | [.14, .26] | [.15, .28] | [-.07, .02] | [-.08, .02] | [-.04, .04] | [-.05, .04] |
| Social identity |  |  |  |  |  |  |  |  |  |
| Identification with course | β | .07^**^ | .07^**^ | -.02 | -.03 | .20^***^ | .24^***^ | .10^***^ | .11^***^ |
|  | 95%CI | [.02, .12] | [.02, .12] | [-.07, .02] | [-.08, .03] | [.15, .26] | [.17, .31] | [.06, .14] | [.06, .16] |
| Identification with classmates | β | .02 | .03 | .03 | .03 | .13^***^ | .16^***^ | .29^***^ | .34^***^ |
|  | 95%CI | [-.03, .08] | [-.03, .09] | [-.02, .08] | [-.03, .09] | [.08, .17] | [.10, .22] | [.23, .34] | [.27, .40] |

*Note*. 95%CI = 95% conﬁdence interval; T = time.

^*^*p* < .05; ^**^*p* < .01; ^***^*p* < .001.

Table S11

Sensitivity analysis for within-time correlations and correlated changes between personal identity and social identity, controlling for majors

| Variables |  | Identity confusion | | | Identification with course | | | Identification with classmates | | |
| --- | --- | --- | --- | --- | --- | --- | --- | --- | --- | --- |
|  |  | T1 | T2 | T3 | T1 | T2 | T3 | T1 | T2 | T3 |
| Personal identity |  |  |  |  |  |  |  |  |  |  |
| Identity synthesis | *r* | -.10^**^ | -.15^***^ | -.11^***^ | .14^***^ | .19^***^ | .17^***^ | .27^***^ | .20^***^ | .18^***^ |
|  | 95%CI | [-.17, -.03] | [-.22, -.08] | [-.17, -.05] | [.08, .20] | [.13, .25] | [.12, .22] | [.21, .33] | [.14, .25] | [.13 .23] |
| Identity confusion | *r* | – | – | – | -.05 | -.12^***^ | -.03 | -.05 | -.12^***^ | -.02 |
|  | 95%CI | – | – | – | [-.11, .01] | [-.18, -.06] | [-.08, .02] | [-.12, .03] | [-.17, -.06] | [-.07, .04] |
| Social identity |  |  |  |  |  |  |  |  |  |  |
| Identification with course | *r* |  |  |  | – | – | – | .39^***^ | .56^***^ | .56^***^ |
|  | 95%CI |  |  |  | – | – | – | [.33, .45] | [.52, .60] | [.52, .59] |
| Identification with classmates | *r* |  |  |  |  |  |  | – | – | – |
|  | 95%CI |  |  |  |  |  |  | – | – | – |

*Note*. 95%CI = 95% conﬁdence interval; T = time.

^**^*p* < .01; ^***^*p* < .001.

Table S12

Associations of majors with personal identity and social identity

| Study variables |  | Majors (0 = things-oriented, 1 = people-oriented) | | |
| --- | --- | --- | --- | --- |
|  |  | T1 | T2 | T3 |
| Personal identity |  |  |  |  |
| Identity synthesis | β | .07^**^ | .02 | -.02 |
|  | 95%CI | [.03, .11] | [-.02, .06] | [-.06, .01] |
| Identity confusion | β | -.09^***^ | -.04 | .01 |
|  | 95%CI | [-.13, -.05] | [-.08, .00] | [-.02, .05] |
| Social identity |  |  |  |  |
| Identification with course | β | .12^***^ | .01 | .00 |
|  | 95%CI | [.08, .16] | [-.02, .06] | [-.04, .04] |
| Identification with classmates | β | .23^***^ | .11^***^ | .09^***^ |
|  | 95%CI | [.18, .27] | [.08, .15] | [.05, .12] |

*Note*. 95%CI = 95% conﬁdence interval; T = time.

^**^*p* < .01; ^***^*p* < .001.

**References**

Chen, F. F. (2007). Sensitivity of goodness of ﬁt indexes to lack of measurement invariance. *Structural Equation Modelling*, *14*(3) 464–504. https://doi.org/10.1080/10705510701301834

Kuhn, A., & Wolter, S. C. (2022). Things versus people: Gender differences in vocational interests and in occupational preferences. *Journal of Economic Behavior and Organization*, *203*, 210–234. https://doi.org/10.1016/j.jebo.2022.09.003

Satorra, A., & Bentler, P. M. (2001). A scaled difference chi-square test statistic for moment structure analysis. *Psychometrika*, *66*(4), 507–514. https://doi.org/10.1007/BF02296192

Su, R., Rounds, J., & Armstrong, P. I. (2009). Men and things, women and people: A meta-analysis of sex differences in interests. *Psychological Bulletin*, *135*(6), 859–884. https://doi.org/10.1037/a0017364
